# Recent Advances in Cyanotoxin Synthesis and Applications: A Comprehensive Review

**DOI:** 10.3390/microorganisms11112636

**Published:** 2023-10-26

**Authors:** Zipeng Li, Xiaofei Zhu, Zhengyu Wu, Tao Sun, Yindong Tong

**Affiliations:** 1 School of Environmental Science and Engineering, Tianjin University, Tianjin 300072, China; zpheidi@163.com (Z.L.); wuzhengyu@tju.edu.cn (Z.W.); 2 Laboratory of Synthetic Microbiology, School of Chemical Engineering & Technology, Tianjin University, Tianjin 300072, China; zhuxiaofei1221@163.com; 3 Center for Biosafety Research and Strategy, Tianjin University, Tianjin 300072, China; 4 College of Ecology and Environment, Tibet University, Lhasa 850000, China

**Keywords:** cyanotoxins, biosynthesis pathway, heterologous biosynthesis, synthetic biology, potential applications

## Abstract

Over the past few decades, nearly 300 known cyanotoxins and more than 2000 cyanobacterial secondary metabolites have been reported from the environment. Traditional studies have focused on the toxic cyanotoxins produced by harmful cyanobacteria, which pose a risk to both human beings and wildlife, causing acute and chronic poisoning, resulting in diarrhea, nerve paralysis, and proliferation of cancer cells. Actually, the biotechnological potential of cyanotoxins is underestimated, as increasing studies have demonstrated their roles as valuable products, including allelopathic agents, insecticides and biomedicines. To promote a comprehensive understanding of cyanotoxins, a critical review is in demand. This review aims to discuss the classifications; biosynthetic pathways, especially heterogenous production; and potential applications of cyanotoxins. In detail, we first discuss the representative cyanotoxins and their toxic effects, followed by an exploration of three representative biosynthetic pathways (non-ribosomal peptide synthetases, polyketide synthetases, and their combinations). In particular, advances toward the heterologous biosynthesis of cyanotoxins in vitro and in vivo are summarized and compared. Finally, we indicate the potential applications and solutions to bottlenecks for cyanotoxins. We believe that this review will promote a comprehensive understanding, synthetic biology studies, and potential applications of cyanotoxins in the future.

## 1. Introduction

Cyanobacteria are autotrophic organisms that utilize light as their energy source to convert CO_2_ into biomass [1]. In recent years, numerous studies have shown that the increasing levels of nitrogen and phosphorus in waters, as well as rising CO_2_ levels in the atmosphere, have led to the rapid growth and proliferation of cyanobacteria [2,3]. As a result, aquatic ecosystems have been severely impacted, with decreased dissolved oxygen levels and disruptions to biochemical cycling and energy flow [3]. Meanwhile, some species of cyanobacteria, such as *Microcystis* sp., *Anabaena* sp., *Cylindrospermopsis* sp., *Aphanizomenon* sp., and *Oscillatoria* sp., produce toxic secondary metabolites called cyanotoxins, which pose a risk to both the ecosystem and human health [4]. Cyanotoxins are typically classified into four types based on the organs that they affect: hepatotoxins, neurotoxins, dermatotoxins, and cytotoxins. Microcystins, which are often produced by freshwater cyanobacteria, are the most widespread and abundant cyanotoxins. Microcystins have diverse structures. In fact, cyanobacterial blooms have caused drinking water crises in Lake Taihu in China and Lake Erie in North America, among other locations [5,6]. In addition, massive wildlife deaths, such as those of elephants and zebras in Botswana and Kruger National Park in South Africa, have been caused by the animals consuming water polluted by cyanotoxins [7,8]. Therefore, the World Health Organization (WHO) has established a guideline value of 1 μg/L for microcystin-LR in drinking water, as well as a short-term exposure threshold of 12 μg/L [9].

Over the past few decades, increasing studies have been carried out focusing on cyanotoxins due to their impacts on the environment and human beings. These studies have encompassed multiple aspects, including chemical structure identification, biosynthesis pathway elucidation, and exploration of toxic effects, influencing factors, and degradation methods [10,11,12]. As of now, there are nearly 300 known cyanotoxins and more than 2000 cyanobacterial secondary metabolites that have been reported from various sources [13], which have been collected in several databases such as Antibase, MarinLit, Cyanomet, and CyanoMetDB [14]. Interestingly, recent studies have shown that these cyanotoxins could also be valuable products in biotechnology and drug development because of their toxic mechanisms [15]. For instance, the microcystins and anatoxin-a have been widely explored as natural insecticides; however, their potential non-target toxicity have hindered the use in commercial products [16,17,18]. Saxitoxin could be employed as a local anesthetic in combination with other drugs to improve the anesthetic effect by blocking nerve channels [19]. Additionally, apratoxin and cryptophycin have exhibited the ability to combat cancer cells [20,21], while lyngbyatoxin could serve as a protein kinase activator by strongly binding and activating protein kinase (PKC) [22,23]. These findings suggest that cyanotoxins may be hidden gems beyond their known hazards.

Actually, the biotechnological potential of cyanotoxins is underestimated, as increasing studies have demonstrated their roles as valuable products, including as allelopathic agents, insecticides, and biomedicines. To promote a comprehensive understanding of cyanotoxins, a critical review is in demand. In this review, we discuss the classifications, biosynthetic pathways, and potential applications of cyanotoxins based on studies mainly from recent years [12,24,25]. Specifically, we first discuss representative cyanotoxins and their toxic effects on humans and animals, followed by an exploration of their biosynthetic pathways. We summarize and compare progress toward the heterologous biosynthesis of cyanotoxins in vitro and in vivo. Finally, we indicate possible solutions to current bottlenecks and the potential for cyanotoxin use. This review aims to promote a comprehensive understanding of cyanotoxins, which will help in the further analysis of toxicological research and provide new insights for the rational valorization of cyanobacteria metabolites.

## 2. Representative Cyanotoxins and Their Toxicology

**Figure 1:**
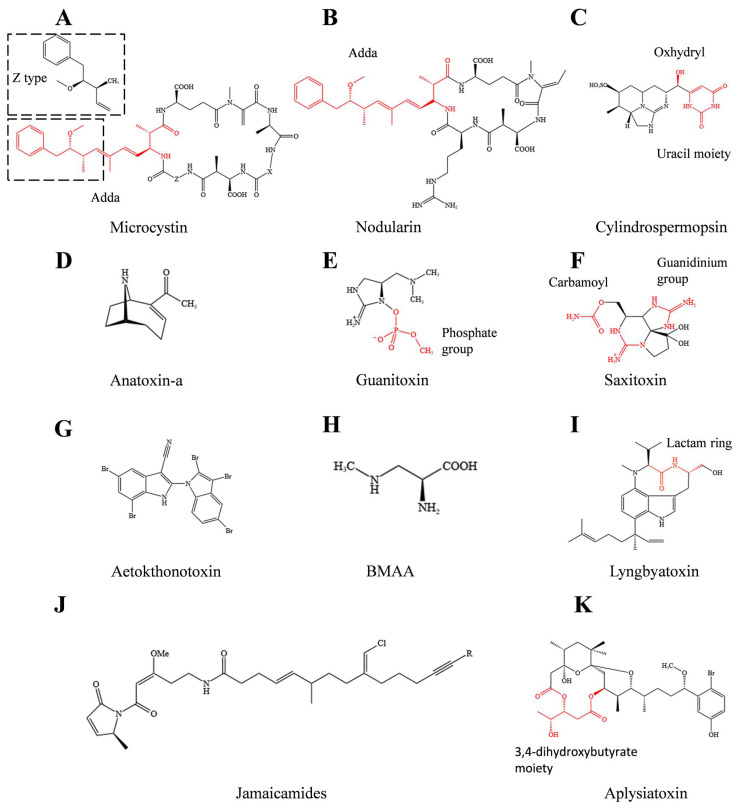
Chemical structural formula of representative cyanotoxins (biological activity is marked in red). (**A**) structure of microcystins, (**B**) structure of nodularin, (**C**) structure of cylindrospermppsin, (**D**) structure of anatoxin-a, (**E**) structure of guanitoxin, (**F**) structure of saxitoxin, (**G**) structure of aetokthonotoxin, (**H**) structure of BMAA, (**I**) structure of lyngbyatoxin, (**J**) structure of jamaicamides, (**K**) structure of aplysiatoxin.

Since the 1990s, there has been growing interest in identifying and categorizing cyanotoxins. Cyanotoxins are classified based on their distinct toxicological effects, including hepatotoxins, neurotoxins, dermatotoxins, and cytotoxins. Table 1 presents a list of representative cyanotoxins for each class, along with their respective toxicities. Chemical structures are illustrated in Figure 1. For readers seeking additional information on other cyanotoxins, specific databases, such as the recently developed CyanoMetDB, are recommended as resources [14].

### 2.1. Hepatotoxins

Microcystins (MCs) are the most widely distributed cyanotoxins in fresh water. They are cycloheptapeptides with the general structures of D-Ala-L-X-D-MeAsp-L-Z-Adda-D-Glu-Mdha (Figure 1A) (X and Z represent the highly variable L-amino acids aspartic acid and dehydroalanine, respectively, with different methylation forms, both of which contribute to the diversities of microcystin molecules) [12]. They are often produced by freshwater cyanobacteria, including species of *Microcystis*, *Aphanizomenon*, and *Dolichospermum*, with more than 300 variants known to date [14,36]. Among these compounds, MC-LR, MC-RR, and MC-YR produced by *Microcystis* and *Anabaena* are the most concentrated variants in the water environment. Chernoff et al. (2020) [37] compared 10 different MC variants toxicity, including MC-LR, MC-LA, MC-YR, and MC-RR. MC-LR and MC-LA were found to be highly toxic in BALB/c mice with oral dosing of 7 mg/kg [37]. Notably, based on animal models, microcystins are highly hepatotoxic and specifically inhibit the activity of protein phosphatase 1 and 2A (PP1 and PP2A), leading to denaturation of cytoskeletal proteins and damage to the cytoskeletal system [37,38]. If exposed to high concentrations of microcystins for a short time, the cellular structure will be destroyed [39]. Long-term exposure to drinking water contaminated with microcystins will cause diarrhea, nerve paralysis, and rapid proliferation of liver cancer cells [40]. The amino acid adda, also known as (2S, 3S, 8S, 9S)-3-amino-9-methoxy-2,6,8-trimethyl-10-phenyldeca-4,6-dienoic acid, is essential for the biological activity of microcystins (Figure 1A). Protein phosphatase inhibition decreases by 10^5^ times when the double bond carbon of C-6 in the adda structure changes to the Z type [41,42] (Figure 1A).

### 2.2. Neurotoxins

Anatoxin-a (ATX-a), also known as very fast death factor (VFDF), is a neurotoxic alkaloid produced by some species of freshwater cyanobacteria, including species from the genera *Aphanizomenon*, *Cylindrospermum*, *Microcoleus*, and *Anabaena*. Due to its structural similarity to the neurotransmitter acetylcholine, anatoxin-a could act as an agonist of the nicotinic acetylcholine receptor. Unlike acetylcholine, anatoxin-a cannot be inactivated by acetylcholinesterase hydrolysis due to its rigid molecular structure, thus leading to muscle cell overexcitation and muscle spasm [43] (Figure 1D). Notably, an acute poisoning death occurred in Wisconsin, United States, in 2002 due to the ingestion of anatoxin-a-rich pool water [44]. In addition, a number of dogs, cattle, and other terrestrial animals suddenly died due to drinking or long-term exposure to water polluted by filamentous cyanobacteria bloom in Europe and the United States [45]. This fact has forced the countries concerned to establish safety limit values for anatoxin-a in drinking water. Based on previous toxicity research on sub-chronic and acute ATX-a, the provisional reference value for ATX is 30 μg/L for acute or short-term drinking water exposure [9]. Anatoxin-a (S), also produced by *Anabaena*, is considered a variant of anatoxin-a due to the similar toxic effect. It is the only known natural organophosphate neurotoxin. The toxin is distinguished by “S” because it causes salivation in mice during poisoning, and it was renamed guanitoxin (GNT) due to its guanidine group [30]. In addition, guanitoxin is about 10 times more toxic than anatoxin-a due to its irreversible binding with the serine residue of acetylcholinesterase. Nevertheless, studies of guanitoxin biosynthesis, poisoning events, and detection methods have remained limited.

Saxitoxin (STX) is another neurotoxin referred to as a paralytic shellfish toxin that is produced by the marine species *dinoflagellates* and some freshwater cyanobacteria (Figure 1F). Saxitoxin features a unique tricyclic chemical structure with two guanidinium moieties that exhibits its toxicity by binding to site 1 of voltage-gated Na^+^ channels, as well as Ca^+^ and K^+^ channels (with low affinity). As a result, it can block neuromuscular conduction processes, causing a series of toxic symptoms, such as nausea, paralysis, and respiratory failure [31,46]. Saxitoxin has been listed in the Chemical Weapons Convention (CWC) with a lethal dose of 540–1000 μg for adults, representing one of the most potent marine toxins [47]. Interestingly, saxitoxin has potential applications in red tide detection, neurobiology, drug development, and military biological warfare agents. In addition, the anesthetic effect of other compounds, such as tricyclic antidepressants (TCAs), was improved when used in combination with saxitoxins [48]. Neosaxitoxin has been used as a local long-lasting analgesic, as well as in veterinary medicine for the treatment of bucked shins pain [49].

Vacuolar myelinopathy is a fatal neurological disease first discovered during a mass mortality of bald eagles in Arkansas in 1994. In the ensuing decades, scientists endeavored to understand its causes. Recently, Breinlinger et al. (2021) [32] demonstrated that the epiphytic cyanobacterium *Aetokthonos hydrillicola* growing on the invasive plant *Hydrilla verticillata* can produce the aetokthonotoxin (AETX) using available bromine. Aetokthonotoxin, which is a pentabrominated biindole alkaloid, can cause vacuolar myelinopathy in avian species, especially eagles. After sequencing the genome of *Aetokthonos hydrillicola*, putative biosynthetic gene clusters of aetokthonotoxin including six genes were identified [32]. Through bioassay of *Ceriodaphniadubia*, *Caenorhabditis elegans*, zebrafish, and avian species, aetokthonotoxin was found to be responsible for multiple neurotoxicity symptoms, such as general convulsions, imbalance, and widespread vacuolization in the white matter of the brain [32]. However, the potential impact of aetokthonotoxin on the environment and humans remains unknown.

### 2.3. Dermatotoxins and Cytotoxins

Unlike hepatotoxins and neurotoxins with acute lethality, dermatotoxins and cytotoxins have highly specific biological activities, rather than lethal effects. Lyngbyatoxin and aplysiatoxin, produced by marine cyanobacteria, cause skin irritation, oral inflammation, and gastrointestinal discomfort after human contact. Dermatotoxins produced by species of *Lyngbya* have caused “swimmer’s itch” events in Okinawa, Japan, and Oahu, Hawaii [50]. In addition, apratoxin A; hantupeptin A, produced by *Lyngbya majuscule*; and cryptophycin, produced by species of *Nostoc*, are potent cytotoxins [51]. These cytotoxins have selective cytotoxicity against polyp cells of the breast and pancreas and may have potential as anti-tumor drugs [52].

## 3. Analysis of Cyanotoxins Biosynthesis Pathway

**Figure 2:**
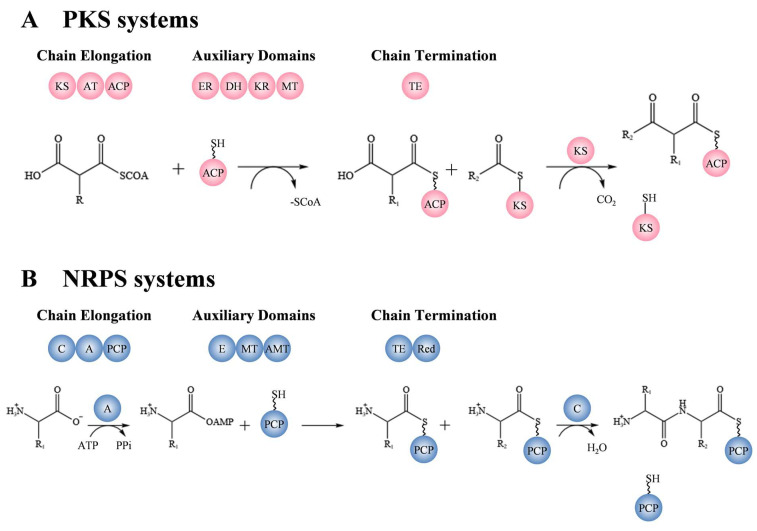
The peptide chain in cyanotoxin synthesis pathways of PKS and NRPS. (**A**) Basic composition of domains in polyketide synthetases. KS, ketosynthase; AT, acyltransferase; ACP, acyl carrier protein; ER, enoylreductase; DH, dehydratase; KR, ketoreductase; MT, methyltransferase; TE, thioesterase. (**B**) Basic composition of domains in non-ribosomal peptide synthetases. C, condensation domain; A, adenylation domain; PCP, peptidyl carrier protein; E, epimerization domain; MT, methyltransferase; AMT, aminotransferase; TE, thioesterase; Red, reductase.

Most cyanotoxins are cyclic compounds with low molecular weights, comprising unusual amino acids and other functional groups [53]. It has been suggested that cyanotoxins are synthesized by non-ribosomal peptide synthetases (NRPSs), polyketide synthetases (PKSs), or both (NRPS/PKS hybrid) [53] (Figure 2). With the aid of genome sequencing, biochemistry, and bioinformatics, researchers have successfully identified the key genes of PKS and NRPS involved in the synthesis of cyanotoxins. For example, LtxA is one of the NRPS related to lyngbyatoxin (LTX) biosynthesis [54], while the AnaE, AnaF, and AnaG are PKSs involved in anatoxin-a production [55,56]. In addition, microcystins, nodularin, cylindrospermopsin, and jamaicamides contain polyketide synthetase–nonribosomal peptide synthetase hybrid clusters (NRPS/PKS) [33,57,58,59].

### 3.1. PKS and NRPS Participating in Cyanotoxin Biosynthesis

The function of PKSs is decarboxylation and Claisen condensation of acyl-CoA, basically including an acyltransferase domain for substrate selection, an acyl carrier protein domain for substrate shuttle and activation, and a ketosynthase domain for Claisen condensation. Meanwhile, the auxiliary domains include the ketoreductase domain for reduction of β-carbonyl to β-hydroxyl, a dehydratase domain for dehydration of β-hydroxyl to α, β-unsaturated olefin, an enoylreductase domain for reduction of olefins to saturated alkanes, and a methyltransferase domain (Figure 2A). The peptide chain undergoes extension through a condensation reaction using malonyl-CoA as a unit, resulting in the formation of a diketone compound. This compound is then modified by one or more auxiliary genes. This process is common in the biosynthesis of anatoxin-a and cylindrospermopsin [53]. SxtA, which is involved in saxitoxin biosynthesis, encodes a unique four PKS-domain enzyme with an 8-amino-7-oxononanoate synthase (AONS) domain, yet it lacks the hallmark ketosynthase domain of PKS [60]. Malonyl-CoA is first bound to the acyl carrier protein domain and further methylated by the methyltransferase domain. The acyltransferase domain then decarboxylates the methylmalonyl-ACP to propionyl-ACP. Finally, a C-C bond is formed between the α-C of Arg and the thioester carbon of acyl-ACP substrate by the PLP-dependent AONS. The single AONS can successfully transform Arg into various ketone derivatives. The study not only demonstrated the potential of the SxtA AONS as a valuable biocatalyst but also presented the concept of synthesizing specific products through domain mutagenesis or medium optimization (e.g., the addition of a methyl ketone) [61].

NRPS synthesize peptides with a variety of structures and biological activities using amino acids as substrates. A standard process includes an adenylation domain for activating the aminoacyl substrate into amino acids, a peptidyl carrier protein domain for bonding the amino acids transferred by the adenylation domain, and a condensation domain for condensing to form peptide bonds. An epimerization domain, an N-methyltransferase domain, and others are used as auxiliary domains (Figure 2B). LtxA, which is an NRPS, begins with the formation of a peptide bond by PCP-bound L-Val and downstream PCP-bound L-Trp under the effect of a condensation domain. L-Val is bound to the amino group of L-Trp. Unlike most NRPSs, LtxA uses reductase to release products [62]. Basic composition of domains in polyketide synthetases and non-ribosomal peptide synthetases and their functions are listed in Table 2.

### 3.2. NRPS/PKS Participating in Cyanotoxin Biosynthesis

**Figure 3:**
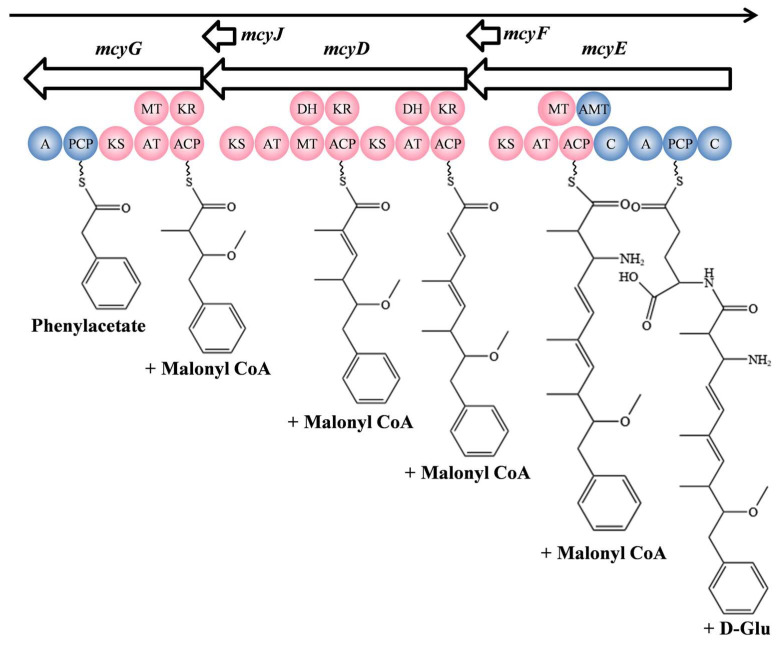
The biosynthesis pathway of adda in microcystins. Composition of domains in adda biosynthesis: KS, ketosynthase; AT, acyltransferase; ACP, acyl carrier protein; DH, dehydratase; KR, ketoreductase; MT, methyltransferase; C, condensation domain; A, adenylation domain; PCP, peptidyl carrier protein; AMT, aminotransferase.

Biosynthesis of the majority of cyanotoxins is catalyzed by the NRPS/PKS hybrid gene clusters. The biosynthesis of microcystin was one of the first NRPS/PKS discovered in bacteria, involving 10 genes (from *mcyA* to *mcyJ*) spanning 55 kb in the genome of *Microcystis aeruginosa* PCC 7806 [70]. Among them, *mcyA*, *mcyB*, and *mcyC* encode NRPS, while *mcyD* encodes PKS, along with *mcyE* and *mcyG* encoding NRPS/PKS hybrids. As stated above, adda is an essential component of the biological activity of microcystins (Figure 1A), and it is synthesized by the genes *mcyD*, *mcyE*, *mcyF*, *mcyG*, and *mcyJ* [70]. To start, McyG contains an adenylation domain and peptidyl carrier protein domain that use phenylpropanoid as a substrate [71]. Then, the chain is bound to the PCP domain, and a molecule of malonyl-CoA is added by PKS. The side chain is modified by methylation by McyJ, which is an *O*-methyltransferase. With the PKS modules of McyD and McyE, three molecules of malonyl-CoA are added, extending the peptide chain. McyE is essential for adda biosynthesis, conferring the cyclization structure and toxicity of microcystins. D-Glu is activated by the NRPS domains of McyE and added to the peptide chain and converted into β-amino acids by the aminotransferase domain. Finally, the initial adda structure is formed [12]. The complete synthesis process of adda is shown in Figure 3.

## 4. Recent Progress in Heterologous Biosynthesis of Cyanotoxins

Previous studies have often utilized freeze-drying, freeze-thawing, or ultrasonication as methods to disrupt cell walls, facilitating subsequent extractions of cyanotoxins through solid-phase extraction or leaching [72,73]. The solvent types, volumes, temperatures, and durations used in leaching methods vary depending on the specific cyanotoxin being extracted [10]. Direct extraction is a common approach for obtaining cyanotoxins. However, issues such as low yield and/or purity are often encountered, which can be attributed to the unculturable nature of certain cyanobacteria, lengthy culture cycles, or inadequate extraction techniques. For de novo chemical synthesis, expensive chemical reagents, harsh reaction conditions, difficult controls on stereoselectivity, and low product yield make it uneconomical for large-scale preparation. Recent studies have focused on biosynthesis by cell-independent enzymatic synthesis using purified enzymes or heterologous biosynthesis of cyanotoxins in non-natural hosts, such as *Escherichia coli*, *Saccharomyces cerevisiae*, *Streptomyces*, and model cyanobacteria [24,74,75,76,77].

### 4.1. Biosynthesis of Cyanotoxins In Vitro 

Aetokthonotoxin has a unique brominated biindole structure and is the first known natural N1-C2’-linked compound with rare nitrile and polybrominated substitutions in nature. Originally, Ricardo et al. used the Somei–Michael reaction as a crucial step to achieve biindole linkage and eventually accomplished the first total synthesis of aetokthonotoxin in five steps with an overall yield of 29% [77]. With elucidated gene clusters of aetokthonotoxin from the genome of *Aetokthonos hydrillicola* [32], Adak et al. [24] completed its biosynthesis in vitro by expressing and purifying each enzyme (from AetA to AetF) in *E. coli*, followed by enzyme catalysis starting with two molecules of tryptophan. AetF is the first characterized single-component flavin-dependent Trp halogenase that can be activated without accompanying reductase partners and a selectively dibrominated indole ring at 5 and 7 of L-Trp. Subsequently, the nitrile functional group is added to the dibrominated indole ring with AetD and metallocofactor Fe(II). 5-bromo-L-Trp loses ammonia pyruvate, and then AetA regioselectivity brominates the 5-bromoindole into 2,3,5-tribromoindole. AetB is a P450 cytochrome with the ability to catalyze aryl coupling reactions. 5,7-dibromo-indole-3-carbonitrile and 2,3,5-tribromoindole are incubated with AetB enzyme to produces aetokthonotoxin. However, the functions of AetC and AetD must be further confirmed [8,77]. The combination of genomic information with in vitro enzymatic characterization not only elucidates the function of each gene or novel enzyme but also develops new methods for the synthesis of cyanotoxins in vitro.

An additional example of cyanotoxin synthesis in vitro is the nine-step enzymatic process for producing guanitoxin from L-arginine. Lima et al. identified the guanitoxin biosynthetic gene cluster in the genome of *Sphaerospermopsis torques-reginae* ITEP-024, which included nine genes from *gntA* to *gntJ* encoding metabolic enzymes and *gntT* encoding a putative transporter [74]. GntB plays an initiating role in the construction of guanitoxin by catalyzing L-Arg to (S)-4-hydroxy-L-Arg. Pre-guanitoxin was successfully obtained by “one-pot synthesis,” adding purified GntCDGE enzymes and predicted *N*-methyltransferase GntF and the cofactors and co-substrates necessary for the reaction. Then, guanitoxin was produced in situ from pre-guanitoxin with GntAIJ. In this process, GntA catalyzes the hydroxylation of cyclic guanidine pre-guanitoxin, while GntI is responsible for phosphorylation of the hydroxyl group. The final step in the process is O-methylation, which is carried out by GntJ and is crucial for the toxic effects of the resulting compound. This process was demonstrated by comparing the inhibitory effects on recombinant human acetylcholinesterase of intermediates synthesized by GntA and GntI with those of guanitoxin [74]. 

**Figure 4:**
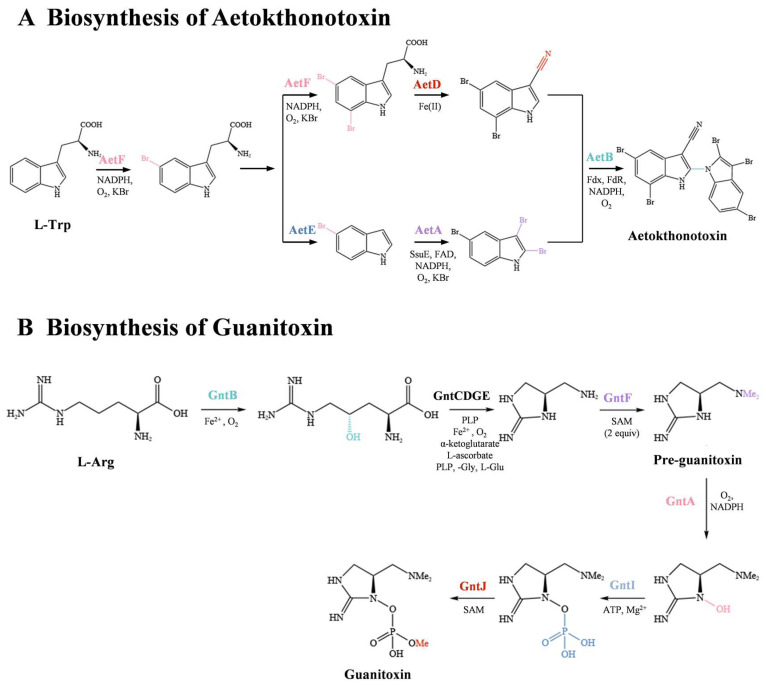
The biosynthesis pathway of aetokthonotoxin and guanitoxin in vitro.

The biosynthesis pathway of aetokthonotoxin and guanitoxin in vitro is shown in Figure 4.

### 4.2. Biosynthesis of Cyanotoxins In Vivo

Transferring gene clusters responsible for cyanotoxin biosynthesis into heterologous microorganisms not only can obtain cyanotoxins but also can allow for creating cyanotoxin analogues or optimizing their yield through gene regulation. *E. coli* is an ideal host for heterologous expression due to its rapid growth and the diversity of tools available [78,79]. Ongley et al. [76] used a gene cluster derived from the cyanobacterium *Moorea producens* for lyngbyatoxin synthesis in *E. coli*, in which the original promoter was replaced with a tetracycline-inducible one. As a consequence, heterologous expression of this pathway achieved high titers of both lyngbyatoxin A (25.6 mg/L) and its precursor indolactam-V (150 mg/L), demonstrating the possibility of expressing other cyanobacterial natural product pathways [76]. Further, Liu et al. [80] utilized a gene cluster derived from the cyanobacterium *Microcystis aeruginosa* PCC 7806 for microcystin synthesis in *E. coli*, in which the original promoter was also replaced with tetracycline-inducible PbiTet. The phosphopantetheinyl transferase (MtaA) was integrated into the *E. coli* genome to further modify the microcystin synthetase. As a result, the heterologous expression of this pathway achieved a comparable yield (65 μg/L) to [D-Asp3]microcystin-LR with natural strains [80]. Furthermore, co-expression of the biosynthetic gene cluster with PPTase, especially Sfp from *Bacillus subtilis*, has been shown to improve the efficiency of heterologous expression of cyanotoxins in *E. coli*. This approach has resulted in further improvements in yield [75].

Given that *E. coli* may lack the necessary precursors for cyanotoxin synthesis, expressing their biosynthetic pathways in model cyanobacteria can be a more effective method. For example, Videau et al. introduced the lyngbyatoxin synthesis *ltxA-C* gene cluster into *Nostoc* sp. PCC 7120 using a transformation-associated recombination (TAR)-capable vector [81]. Interestingly, the new host *Nostoc* sp. PCC 7120 recognized the original promoter in the *ltxA-C* gene cluster, enabling the production of lyngbyatoxin comparable to the natural producer *Moorea producens* but without the accumulation of the precursors ILV and NMVT. Notably, replacing the native promoter *ltxA* with the heterologous promoters P*petE*, P*nirA*, and P*glnA* could increase the yield of lyngbyatoxin A. In addition, analogues, including pendolmycin and teleocidin, were produced, respectively, using the indolactam and codon optimized genes *ltxAB-MpnD* and *ltxABC-TleD* from *Actinomyces* sp., B-4 in *Nostoc* sp. PCC 7120 [82]. More recently, Zheng et al. [25] recombined the *mcy* gene cluster for microcystin biosynthesis driven by a strong bidirectional promoter biPsbA2 in the model cyanobacteria *Synechococcus elongatus* PCC 7942 and successfully obtained MC-LR with production of 0.006–0.018 fg/cell·day. In addition, the research further demonstrated that the expression of MC-LR in *Synechococcus elongatus* PCC 7942 caused abnormal cell division and cell filamentation due to the altered binding of FtsZ (assembled Z-ring structure) to GTP [25]. It was the first reported heterologous synthesis of MC-LR in photosynthetic model organisms, providing new opportunities for studying the effects of cyanotoxins on other hosts.

## 5. Potential Applications of Cyanotoxins

**Figure 5:**
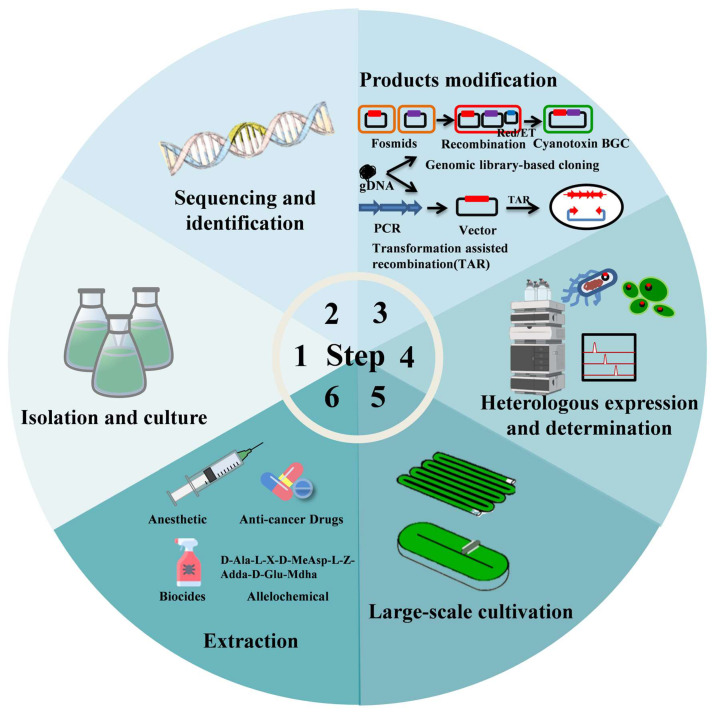
Potential applications and related pathways of cyanotoxins.

Compounds often exhibit dual properties, which can pose a risk to both the environment and human health but can also be utilized to develop valuable products. While the serious ecological hazards of cyanotoxins have been widely acknowledged, their specific modes of toxicity also offer the potential for the development of high-value products. Cyanotoxins have been effectively utilized as allelochemicals, insecticides, antitumor drugs, anesthetics, and other applications [10].Potential applications and related pathways of cyanotoxins can be found in the Figure 5.

### 5.1. Allelopathic Agents and Biocides

A number of secondary metabolites produced by cyanobacteria inhibit the growth of large land plants, algae, and microorganisms, making them potential allelochemicals. MC-LR and MC-RR have been demonstrated to inhibit photoautotrophic organisms, affecting the production of photosynthetic pigments and the activity of PSII in cyanobacteria [83,84,85]. They also inhibit the growth of *Nostoc*, *Synechococcus*, *Anabaena*, and *Chlorophyta*, while enhancing the adaptability of survival cells as cell signaling molecules [86]. The synthesis of BMAA by cyanobacteria is considered a stress response to nitrogen limitation. It inhibits the growth of cyanobacteria without the ability to produce BMAA by affecting the nitrogen assimilation pathway and stimulates the accumulation of BMAA in diatoms [87]. MC-LR and ATX-a also have inhibitory effects on insect larvae, such as the stem borer *Chiloagamemnon* and the leaf miner *Hydrellia prosternalis*, and they can be used as insecticides [18]. However, their high cytotoxicity and poisoning effects on human and animals and the potential non-target toxicity may hinder their use in commercial products [44]. It is crucial to consider the potential adverse effects of modified natural cyanotoxins on non-target organisms when designing candidate agricultural product. While there is potential for valuable product development, it is essential to ensure that any cyanotoxins or other secondary metabolites do not unintentionally harm the environment or non-target organisms.

### 5.2. Biomedicines

Cyanotoxin analogues function as potential antitumor agents. Certain cyanotoxins exhibit strong cytotoxicity to cancer and viral cells, making them potential antitumor agents [88]. Apratoxin A shows strong antitumor activity that arrests cancer cells in the G1 phase and induces an apoptosis cascade [89]. Luesch et al. (2006) synthesized apratoxin analogues through amino acid substitution and discovered that apratoxin A/E hybrids had strong antitumor activity and selectivity, thus improving the selectivity of apratoxin A for cancer cells [90]. The selectivity of cyanotoxins for specific substrates in the secretory pathway can be modulated through structural modifications. They can be used for targeted cancer therapy. Cryptophycin exhibits excellent antifungal and selective antitumor abilities, mainly causing apoptosis of cancer cells by blocking microtubule formation. In detail, its function mechanism seems like that of Vinca alkaloids, hindering the formation of mitotic spindles during the cell cycle, thus leading to mitotic arrest [91]. Its antitumor ability is hundreds of times stronger than that of existing antitumor drugs [92].Cryptophycin-52 not only has microtubule inhibition activity but also showed the ability to induce apoptosis by Bcl-2 phosphorylation in the human H460 non-small-cell lung carcinoma (NSCLC) cell line [93]. Unfortunately, it failed in phase II clinical trials because of its immeasurable responses and a significant level of neurotoxicity [94]. Notably, cryptophycin has shown its potential use in targeted therapeutic approaches. It shows no toxicity, improved stability in plasma, and highly selective anticancer effects in the recent development of small molecule–drug conjugates (SMDCs) [95,96] Other cyanobacterial bioactive compounds, such as dolastatin 10, calothrixin A, and symplocamide A, inhibit cancer cell growth by affecting the cell cycle, inducing mitochondrial rupture, oxidative damage, altering apoptotic signaling pathways, regulating caspase signal transduction, or controlling Na^+^ channels [97,98,99]. Lyngbyatoxin A has been shown to possess anti-HIV properties, while aplysiatoxins exhibit promising anti-Chikungunya-virus (CHIKV) activity. Currently, the use of microalgae as a vaccine production host and delivery vehicle is becoming a mature method [100]. It could be considered to purify cyanotoxins with antiviral ability into injectable antigens for stimulating the immune system and reducing the use of antibiotics in the case of illness. Intensive studies of the potential anticancer drug usage of cyanobacteria products have mostly focused on other cyanobacterial bioactive compounds with low toxicity. However, based on these enlightening, creative studies, more efforts could focus on how to explore their biological activity.

Cyanotoxin analogues exhibit the abilities of anesthetics. Cyanotoxins with severe neurotoxicity, such as STX, could suspend nerve function and have great potential for making anesthetics. However, it is important to ensure that the damage to the nerve function is recoverable and does not cause permanent damage. These bioactive cyanobacterial secondary metabolites have great potential to be developed into novel drugs. Dolastatin 10 and cryptophycin have achieved positive results in clinical trials. More detailed information about cyanobacterial secondary metabolites for drug development can be found in the latest review articles [101,102].

## 6. Conclusions and Perspectives

This article provides an overview of cyanotoxins, including their structural formulas, toxin classes, and modes of action. It also highlights the biosynthetic pathways of different types of cyanotoxins, including PKS, NRPS, and PKS/NRPS, as well as recent advancements in heterologous synthesis techniques. Furthermore, the article investigates potential applications for cyanotoxins based on their toxic mechanisms. To deepen our understanding and utilization of cyanotoxins, future studies should focus on clarifying their biosynthesis pathways, overcoming challenges in large-scale purification, and developing strategies to mitigate their toxic effects.

(i) *Accelerating the annotation of biosynthetic pathways for cyanotoxins.* The advent of high-throughput DNA sequencing technologies, coupled with convenient genome mining, has enabled researchers to rapidly identify biosynthesis gene clusters of cyanotoxins in cyanobacteria. Third-generation sequencing technology could provide greater in-depth sequencing, improving the quality of gene-splicing sequences. Isotopic tracing and transcriptomics also could be applied to validate the genetic pathway. Finally, functional gene annotation could be verified through in vitro experiments.

(ii) *Developing effective production strategies for cyanotoxins.* Natural hosts are not convenient for large-scale production of cyanotoxins due to their slow growth rate and/or uncultivability. Alternatively, heterologous expression systems for cyanotoxins in microorganisms such as *E. coli* could be more effective. However, limitations, such as cloning and assembling large DNA fragments and low or silent gene expression in heterologous hosts, still hinder heterologous expression. Cloning large biosynthetic gene clusters with high repeatability and high GC content is a challenging and critical step for heterologous expression. In the future, in vitro assembly technology based on CRISPR/Cas, developed especially for large fragments, could be considered [103]. Meanwhile, other hosts in addition to *E. coli* could be tested.

(iii) *Modifying natural cyanotoxins to regulate their toxic effects and make high-value products.* Medicinal chemistry and synthetic biology methods could be used to optimize the biosynthetic gene cluster, which can decrease the toxicity of the product or enhance its specificity of action. Consequently, additional research on cyanotoxins could yield benefits, not only in reducing the harmful effects of cyanotoxin release on human health and the environment during cyanobacterial blooms but also in the comprehensive utilization of cyanotoxin bioactivity to produce high-value products.

## Figures and Tables

**Table 1:** Examples of representative cyanotoxins.

Classifications	Toxin	Compound Type	Toxicology	Reference
Hepatotoxins	Microcystin	Cyclic heptapeptide	Inhibition of eukaryotic protein phosphatases	[26]
	Nodularin	Cyclic pentapeptide	Inhibition of eukaryotic protein phosphatases	[27]
	Cylindrospermopsin	Guanidine alkaloid	Inhibition of protein synthesis, DNA damage, and genotoxicity	[28]
Neurotoxins	Anatoxin-a	Alkaloid	Agonist of nicotinic acetylcholine receptors	[29]
	Guanitoxin	Organophosphate	Irreversible inhibition of acetylcholinesterase	[30]
	Saxitoxin	Tricyclic alkaloid	Block voltage-gated sodium channels of neurons; inhibition of acetylcholinesterase activity	[31]
	Aetokthonotoxin	Pentabrominated biindole alkaloid	Unknown	[32]
	BMAA *	Nonproteinogenic amino acid	Agonist of glutamate receptors, association with proteins, induction of oxidative stress	[2]
	Jamaicamides	Polyketide–peptide	Sodium channel-blocking activity	[33]
Dermatotoxins	Lyngbyatoxin	Indole alkaloid	Protein kinase C activator	[34]
	Aplysiatoxin	Polyketide–peptide	Inhibition of voltage-gated potassium channel activity	[35]

* BMAA, *β*-*N*-methylamino-*L*-alanine.

**Table 2:** Basic composition of domains in polyketide synthetases and non-ribosomal peptide synthetases.

Domain	Abbreviations	Functions	References
PKS systems			
Acyl carrier protein	ACP	Substrate shuttle and activation	[63]
Acyltransferase	AT	Substrate selection	[63]
Dehydratase	DH	Dehydration of β-hydroxyl to α, β-unsaturated olefin	[63]
Enoylreductase	ER	Reduction of olefins to saturated alkanes	[63]
Ketoreductase	KR	Reduction of β-carbonyl to β-hydroxyl	[63]
Ketosynthase	KS	Claisen condensation	[63]
Methyltransferase	MT	Tailoring enzyme	[63]
Thioesterase	TE	Thiol group hydrolyzation, product release	[62,64]
NRPS systems			
Adenylation domain	A	Aminoacyl substrate activation	[65,66]
Aminotransferase	AMT	Catalyzing the redistribution of nitrogen between amino acids and corresponding oxoacids	[67,68]
Condensation domain	C	Condensation	[53]
Epimerization domain	E	Catalyzing the conversion of L-amino acids into D-amino acids	[69]
Methyltransferase	MT	Tailoring enzymes	[66]
Peptidyl carrier protein	PCP	Amino acid bonding	[66]
Reductase	Red	Catalyzing the substrate for hydrogenation	[66]
Thioesterase	TE	Peptide product release	[62,66]

## Data Availability

No new data were created in this study.

## Abbreviations

Acyl-CoA: Acyl coenzyme A; Adda: (2S, 3S, 8S, 9S) 3-amino-9-methoxy-2,6,8-trimethyl-10-phenyldeca-4,6-dienoic; AETX: Aetokthonotoxin; Ala: Alanine; AONS: 8-amino-7-oxononanoate synthase; Arg: Arginine; ATX-a: Anatoxin-a; BMAA: β-N-methylamino-L-alanine; BGC: Biosynthetic gene cluster; CHIKV: Chikungunya virus; D-MeAsp: D-erythro-β-methylaspartic; *E. coli*: Escherichia coli; FAD: Flavin adenine dinucleotide; Fdx: Ferredoxin; FdR: Ferredoxin reductase; FtsZ: Filamentous temperature-sensitive protein Z; Glu: Glutamic acid; GNT: Guanitoxin; ILV: Indolactam V; L-Trp: L-tryptophan; LTX: Lyngbyatoxin; L-Val: L-valine; malonyl-CoA: Malonyl coenzyme A; MC-LR: Microcystin-LR; MC-RR: Microcystin-RR; MCs: Microcystins; MC-YR: Microcystin-YR; Mdha: N-methyldehy droalanine; MtaA: Promiscuous phosphopantetheinyl transferase; NMVT: N-methyl-L-valyl-L-tryptophanol; NRPS: Non-ribosomal peptide synthetases; PKC: Protein kinase C; PKSs: Polyketide synthetases; PLP: Pyridoxal 5′-phosphate; PPTase: phosphopantetheinyl transferases; PSII: Photosystem II; SAM: S-adenosylmethionineSfp: Siderophore synthetase; STX: Saxitoxin; TAR: Transformation-associated recombination; TCAs: Tricyclic antidepressants; Trp halogenase: Tryptophan halogenase; VFDF: Very fast death factor; 5-bromo-L-Trp: 5-bromo-L-tryptophan.
